# Bottom–Up Electrodeposition of Large-Scale Nanotwinned Copper within 3D Through Silicon Via

**DOI:** 10.3390/ma11020319

**Published:** 2018-02-23

**Authors:** Fu-Long Sun, Zhi-Quan Liu, Cai-Fu Li, Qing-Sheng Zhu, Hao Zhang, Katsuaki Suganuma

**Affiliations:** 1 Institute of Metal Research, Chinese Academy of Sciences, Shenyang 110016, China; flsun12s@imr.ac.cn (F.-L.S.); licf@alum.imr.ac.cn (C.-F.L.); 2 University of Chinese Academy of Sciences, Beijing 100049, China; 3 Institute of Scientific and Industrial Research, Osaka University, Osaka 5670047, Japan; zhanghao@eco.sanken.osaka-u.ac.jp (H.Z.); suganuma@sanken.osaka-u.ac.jp (K.S.)

**Keywords:** large-scale electrodeposition, nanotwinned copper, through silicon via, gelatin adsorption

## Abstract

This paper is the first to report a large-scale directcurrent electrodeposition of columnar nanotwinned copper within through silicon via (TSV) with a high aspect ratio (~4). With this newly developed technique, void-free nanotwinned copper array could be fabricated in low current density (30 mA/cm^2^) and convection conditions (300 rpm), which are the preconditions for copper deposition with a uniform deep-hole microstructure. The microstructure of a whole cross-section of deposited copper array was made up of (111) orientated columnar grains with parallel nanoscale twins that had thicknesses of about 22 nm. The hardness was also uniform along the growth direction, with 2.34 and 2.68 GPa for the top and bottom of the TSV, respectively. The gelatin additive is also first reported hereas a key factor in forming nanoscale twins by adsorbing on the cathode surface, in order to enhance the overpotential for cathodic reaction during the copper deposition process.

## 1. Introduction

Three-dimensional integrated circuitry (3D-IC) with through silicon via (TSV) is a promising technology for obtaining vertical electric interconnections [1,2]. A stacked die package through TSV can enable a higher packaging density, faster signal transmission, and lower power consumption. The filling of a conductive material into the micro via is one of the core technologies for determining the performance of TSV. Filling through Cu electroplating is commonly used for its merits of good conductivity and low cost. For the TSV with a high aspect ratio, one effort in the Cu electroplating is toward avoiding voids formation in the filled structure. In addition, the other effort is aimed at fabricating a fine Cu grain structure in order to obtain a 3D interconnect with excellent mechanical, electrical, and thermal properties.

Lu et al. synthesized nanotwinned copper film using pulsed current plating [3]. The nanotwinned copper was reported to exhibit both superior conductivity and high strength [3,4,5,6]. The twin-modified grain boundaries could slow down the electro migration-induced atomic diffusion by one magnitude [7]. Unidirectional intermetallic compound (IMC) growth and no Kirkendall voiding were observed at the Cu/Sn interface after reflow and a thermal aging test, in comparison with the result using twin-free copper as the reflow substrate [8,9,10,11]. A large amount of attention has been given to the high performance of the nanotwinned Cu in microelectronic fabrication. It is expected that an advanced 3D interconnect can be obtained by means of the filling of nanotwinned copper into the TSV. To date, only Xu et al. reported that nanotwinned copper was achieved within TSV by using pulsed current deposition [12]. However, the obtained nanotwinned structure only distributed at the bottom of the micro via, and accounted for a low percentage. The challenge for this technology is to ensure that both a high-density and uniform nanotwinned structure can be obtained in the case of a bottom–up filling mode within the TSV.

Hsiao et al. and Liu et al. electroplated nanotwinned copper to fabricate the under ball metallization (UBM) on wafer, where a strict plating condition of strong convection and high current density needed to be satisfied [8,11]. Unfortunately, the strong convection and high current density may fail to create a bottom–up filling for the TSV with a high aspect ratio. 

This work reports a new direct current electrodeposition technique to fill TSV at a high quality. A large-scale nanotwinned copper filling within the TSV was fulfilled using a gelatin additive in the plating formula by a bottom–up growth mode. The microstructure and mechanical performance of the filled micro via with nanotwinned Cu were characterized. The formation mechanism of the nanotwin and bottom–up filling was investigated by means of an electrochemical analysis.

## 2. Experimental

**Figure 1:**
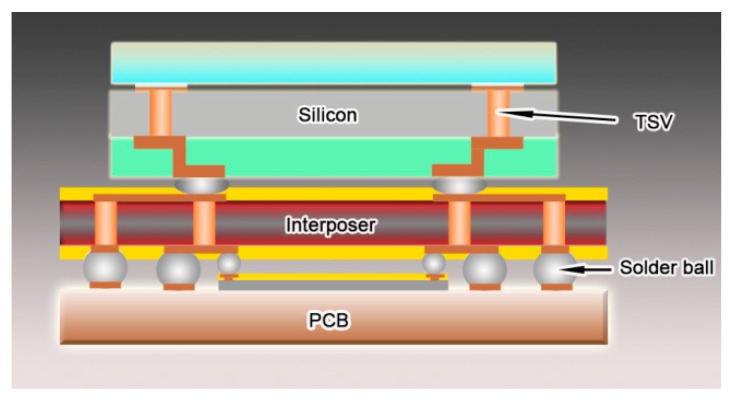
The illustration of through silicon via in electronic component.

A bottom–up direct-current electrodeposition technique was developed to electroplate columnar nanotwinned copper in through silicon via, which has a diameter of 50 μm and a depth of 200 μm. The structure of through silicon vias was illustrated in Figure 1. First, a contact substrate with a 200 nm thick gold seed layer was bonded on the wafer with TSVs as the cathode. An electrolytic copper sheet was put into the bath as the anode. Afterwards, the electrodeposition cell was put into a tank to pump out the remaining gas in deep holes and make electrolyte flow into every hole for deposition. The gas pumping should last more than five hours, so that every hole can be filled with bath; this is the key point in fabricating void-free copper arrays. The bath contains 75 g/L CuSO_4_ solution and 3 mg/L sulfuric acid. First, 5 mg/L gelatin additive was used to produce (111)-orientated nanoscale twins in each copper grain. This concentration is the lowest value for growing coherent twinning boundaries in columnar grains, and the maximum value of the additive in our work should be 70 mg/L. A low concentration may result in incoherent twin boundaries or no nanoscale twins, while a too high concentration could lead to equiaxed grain formation in the deposit, which does not contain nanoscale twin boundaries either in our observation. The electrolyte was agitated by a magnet bar at a low stirring speed of 300 rpm, which could guarantee the uniform microstructure in the growth direction of copper array. The cathodic current density here was 30 mA/cm^2^. The precise control of the growth behavior of copper grains in through silicon vias was first reported in fabricating nanotwinned copper in microelectronic industries, which would certainly advance its application in the electrodeposition of nanotwinned Cu interconnects with complex shapes [8,12]. 

The cross-section copper was observed with scanning electron microscopy (SEM, FEI Quanta 600, FEI, Hillsboro, OR, USA), and a transmission electron microscopy (TEM, JEOL JEM2100, JEOL Ltd., Tokyo, Japan), while the EBSD (Electron Backscattered Diffraction) (Aztec HKL, Oxford Instruments, Oxfordshire, UK) technique was adopted to characterize its crystalline orientation. 

A nanoindentation test was conducted on the cross-section of copper [13]. The electrochemical behavior was measured at an electrochemical working station (Autolab 204, Metrohm, Herisau, Switzerland) equipped with a rotating ring disk electrode (RDE).

## 3. Results and Discussion

**Figure 2:**
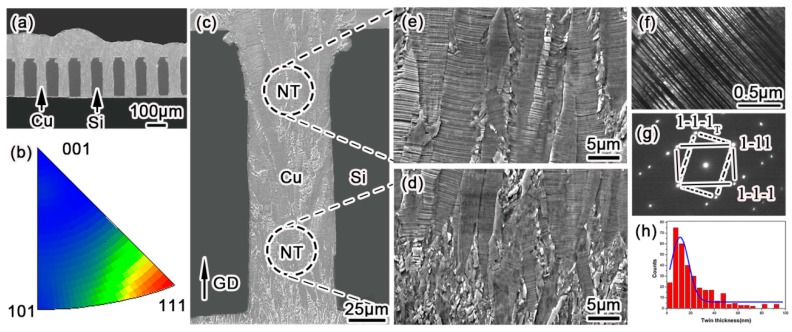
The microstructure of nanotwinned Cu-filled through silicon via (TSV) electroplated with 30 mA/cm^2^ and 300 rpm. (**a**) Void-free plating of Cu in TSV array before surface CMP (Chemical Mechanical Polishing); (**b**) EBSD grain orientation mapping of <111>_Cu_ along the growth direction; (**c**) columnar grain morphology with lamellar striation in one via; (**d**) enlarged bottom and (**e**) top microstructure of TSV with a high density of nanotwins; (**f**) Dark-field TEM image of nanotwinned structure, and (**g**) the corresponding [110]_Cu_ diffraction pattern, where spots from the matrix and the twin lamella are denoted by solid and dashed lines, respectively; (**h**) Histogram of twin thicknesses derived from edge-on TEM observations.

The through-hole filling morphology and microstructural characterization are shown in Figure 2. Figure 2a shows a bottom–up void-free filling of through silicon vias at an aspect ratio of 1:4. The high throw power (TP) in an electrolyte formula with a low concentration of CuSO_4_ and a high amount of H_2_SO_4_ could diminish void formation in a deep microvia. This void-free filling effect was highly uniform over the entire silicon unit, consisting of 136 microvia arrays. The filled Cu within the microvias exhibited a strong <111> crystalline orientation along the growth direction (Figure 2b), which was consistent with the results reported by Hsiao et al. [8], Liu et al. [11], You et al. [14,15], and Pan et al. [16]. Since copper atoms prefer to be reduced on a low surface energy plane, and the (111) plane corresponds to the least surface energy in face-centered cubic (*fcc*) metal, the Cu atoms are prone to deposit on a (111) plane, resulting in a pronounced <111> crystalline orientation along the deposition direction. Figure 2c shows the detailed cross-sectional microstructure of plated copper within one via, in which the “NT” marked zones were magnified, as shown in Figure 2d,e respectively. As a result of the competitive growth among adjacent grains, according to the nucleation–coalescence growth mechanism, the large columnar dendrites were composed of highly aligned twin lamellas, which predominantly paralleled the substrate plane. According to TEM observations, these lamellas are twinning structures, whose dark field image is shown in Figure 2f with the corresponding electron diffraction pattern in Figure 2g. Along this [110]_Cu_ orientation, all of the (111)_Cu_ twinning planes are edge-on. Thus, the twin thickness could be measured accurately from more than 500 twin lamellas, as shown in Figure 2h. The thickness of the coherent twin lamella ranges from several to 100 nanometers with Gaussian distribution, and the average value of the twin thickness was calculated to be about 22 nm, which was comparatively smaller than those reported by others [8,11,12,14,15,16]. This plating result is consistent with our previous work [17]. Series of observations demonstrated that nanotwinned (NT) Cu lamellas could uniformly cover the entire filled microvia at a rather high density. Therefore, through the present direct current electroplating method, the TSV (diameter 30~50 μm, aspect ratio ~4) can be filled with void-free nanotwinned Cu at a large scale.

**Figure 3:**
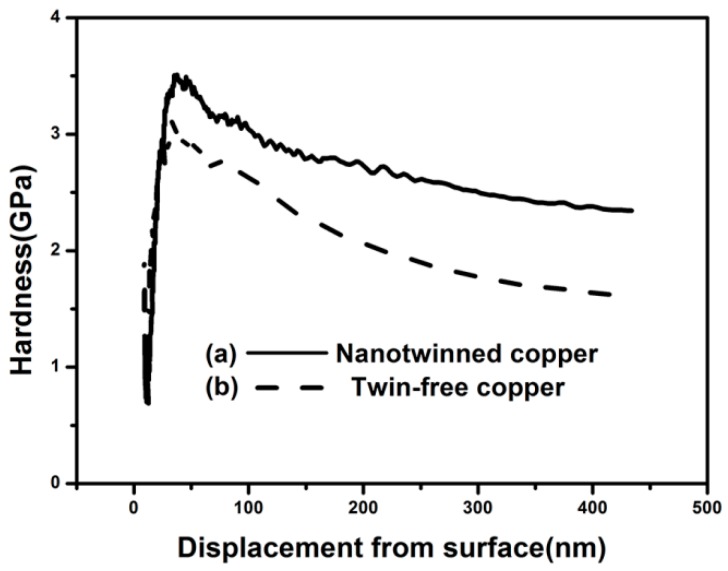
The measured hardness vs displacement from the surface for (**a**) a nanotwinned copper pillar and (**b**) a twin-free copper pillar on the cross-section of the TSV.

The nanoindentation tests were performed on the cross-section of the nanotwinned copper in vias from the TSV, using the same method as reported in the literature [12]. Both the top and the bottom sides were tested to examine the hardness uniformity throughout the entire filled Cu pillar, using a conventional electroplated twin-free Cu pillar for comparison, as shown in Figure 3. The hardness was measured to be 2.34 GPa at the top, and 2.63 GPa at the bottom, respectively, for the nanotwinned copper pillar (see Figure 3a), which is significantly higher than the hardness of 1.73 GPa for the plated copper without nanotwins (see Figure 3b). The hardness difference between the top and bottom side of nanotwinned Cu TSV is very small (0.29 GPa). This verifies the uniformity of the filled nanotwinned Cu pillar, and is also consistent with their similar microstructures, as shown in Figure 2d,e. In another study [12], although a nanoscale twin lamella was formed at the bottom of the filled TSV, they were hardly found at the top of the TSV. The corresponding hardness was also not uniform, decreasing from 2.4 GPa at the bottom to 1.6 GPa at the top. A uniform microstructure and the mechanical properties of the filled nanotwinned Cu pillar reported in this work could reduce the current and heat-induced stress within the TSV during the component’s service, and thus increase the reliability of the devices.

**Figure 4:**
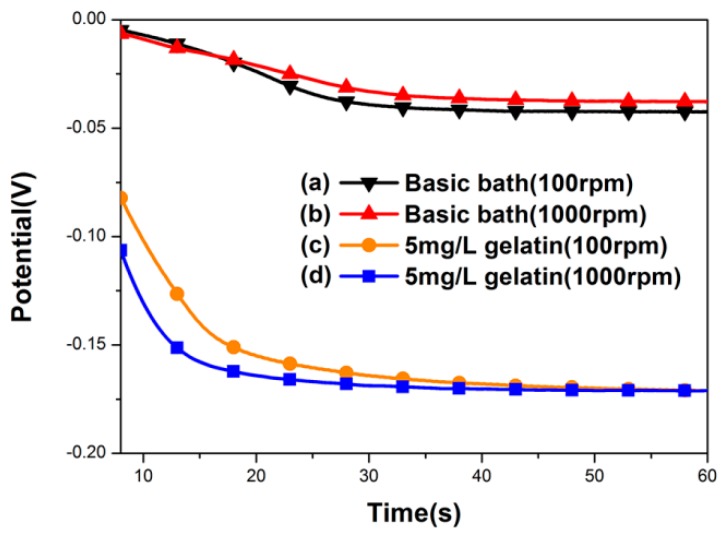
Chronopotentiometry curve for (**a**,**b**) a basic bath, and (**c**,**d**) a gelatin-added bath to produce nanotwinned copper with different stirring rates.

**Figure 5:**
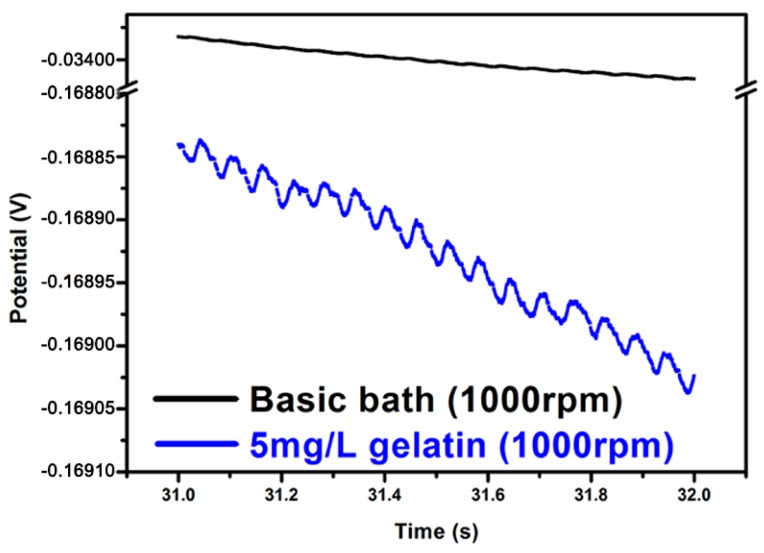
Magnification of the chronopotentiometry curves for a bath with gelatin and a basic bath. Both are stirred at 1000 rpm.

The addition of gelatin was found to be essential for the formation of a nanotwinned Cu structure within the TSV. As shown in Figure 4, the chronopotentiometry curves of the electrolytes with and without gelatin additives were compared with relatively high and low rotation speeds of the RDEs. Using a basic bath without gelatin (see Figure 4a,b), no nanoscale twins were formed in the filled TSV. The electrolytes with gelatin (Figure 4c,d) showed much more negative potential than the electrolytes without gelatin. It suggested that the adsorption of the gelatin on the cathode surface can greatly enhance the overpotential of the copper deposition. Thus, the copper ionic discharge can be suppressed, and even interrupted, when the coverage of the gelatin molecule on the cathode surface is high enough. Following that, a larger negative potential on the cathode can be responded when the deposition is controlled under a constant current mode. When the responding potential reaches a value to desorb the gelatin from the cathode, the deposition will be triggered again. This could be illuminated by the magnified graph of chronopotentiometry curves in Figure 5. Here, we choose a basic bath and a bath with gelatin at 1000 rpm to observe carefully. The representative deposition time of the magnified zone ranges from 31 to 32 s, because in this region, the polarization behavior has become stable, and the polarization curve could be detected in detail. From Figure 5, we can find obvious potential oscillations in a bath with 5 mg/L gelatin, as indicated by the blue line, which corresponds to the process of gelatin desorption and copper redisposition, while in a basic bath, as indicated by the dark line, this oscillation effect is not so significant. In this process, the duration time for the interruption of the deposition corresponds to the offtime in a pulse current deposition. In a short duration time, a short-range atomic rearrangement can be allowed for releasing the stress in the deposited film, while a nanotwinned boundary tends to form in order to reach the lowest energy state. This periodic spontaneous disruption caused by the adsorption and desorption of additives can produce a larger-scale and more uniform nanotwinned Cu structure within the microvia. It was regarded that both on and off time in plating could produce a high level of stress that could be released dramatically by forming twins, which was consistent with what Xu et al. reported in fabricating nanotwinned copper using pulsed current deposition [18]. 

It was reported that the high convection conditions on a copper film surface might displace the Cu atom from its original *fcc* position, resulting in the formation of a high density of twins [8,11]. However, the stirring rate we used here was very low (300 rpm), and the electrolytes at the rotation speeds of both 100 and 1000 rpm exhibited similar potentials, as shown in Figure 4. It demonstrated that the Cu deposition at a deeper location may be the same as that at the shallower location within the TSV. By this bottom–up deposition mode, the early entrance closure that resulted from the current crowding effect can be delayed. Thus, the defects of the voids or seams can be effectively avoided in the filled Cu. In addition, compared to the methods depending on the high current density, the application of the low current density in the present method can also decrease the risk of the void occurrence, and improve the uniformity of the microstructure. 

## 4. Conclusions

A new technique was developed to fill high aspect ratio TSVs from the bottom to the top using direct current electrodeposition. The remaining gas bubbles in deep holes should be pumped out for a void-free deposition. The low stirring rate of the bath could make the convection condition of the TSV bottom and TSV top consistent, facilitating copper deposition in complex-shaped components. Nanotwinned copper was successfully electroplated on a large scale in through silicon vias with a low current density (30 mA/cm^2^) and a low rotation speed (300 rpm) in a bath containing gelatin additives. The filled microstructure is quite uniform, containing (111) orientated columnar copper grains with nanoscale twins (with a lamella thickness of 22 nm) in parallel with the substrate surface. The hardness was uniform from the bottom to the top of the TSV, reaching up to 2.63 GPa. A high mechanical property could make nanotwinned copper serve as framework to prevent wafer warpage when undergoing the wafer-thinning process later.

An electrochemical analysis demonstrated that the gelatin played a key role in forming nanoscale twins by adsorbing on the cathode surface and increasing its overpotential. The deposition follows a bottom–up growth process with a good uniformity of microstructure and hardness, which can enable a good performance of the filled TSV during packaging. This deposition process could be applied in other interconnect fabrications such as UBM (under bump metallization), RDL (redistribution layer), and Copper pillar bump. Applications within these fabrications could lead to a greater property enhancement of packaging material, and thus provide considerable industrial value.
